# Prevalence of group B streptococcus among pregnant women and newborns at Hawassa University comprehensive specialized hospital, Hawassa, Ethiopia

**DOI:** 10.1186/s12879-019-3859-9

**Published:** 2019-04-16

**Authors:** Musa Mohammed Ali, Yimtubezinash Woldeamanuel, Daniel Asrat Woldetsadik, Tolossa Eticha Chaka, Demissie Assegu Fenta, Muluwork Tefera Dinberu, Eskinder Kebede Weldetensaye, Samson Jamal Ismael, Birkneh Tilahun Tadesse

**Affiliations:** 1 School of Medical laboratory science, College of medicine and health science Hawassa University, Hawassa, Ethiopia; 2 Department of Microbiology, Immunology and Parasitology College of Health Science Addis Ababa University, Addis Ababa, Ethiopia; 3 Adama Hospital Medical College, Adama, Ethiopia; 4 Department of Pediatrics, College of Health Science, Addis Ababa University, Addis Ababa, Ethiopia; 5 Department of Gynecology and Obstetrics, College of Health Science, Addis Ababa University, Addis Ababa, Ethiopia; 6 Department of Gynecology and Obstetrics, College of Medicine and Health Sciences, Hawassa University, Hawassa, Ethiopia; 7 Department of Pediatrics, College of Medicine and Health Sciences, Hawassa University, Hawassa, Ethiopia

**Keywords:** Group B streptococcus, Vertical transmission, Prevalence of GBS, Serotype, Hawassa, Ethiopia

## Abstract

**Background:**

Group B streptococcus (GBS) is reported as the leading cause of neonatal sepsis and meningitis. Newborns from GBS colonized pregnant women are at high risk of infection.

**Method:**

A Hospital based cross-sectional study was conducted at Hawassa University Comprehensive Specialized Hospital from November 05, 2014 to March 25, 2015. A total of 280 pregnant women along with their newborns were screened for GBS using standard method recommended by Center of Disease Control and Prevention. GBS strains were serotyped by using serotype specific antisera. A structured questionnaire was used to collect sociodemographic, obstetrics and clinical data of pregnant women and newborns. Data was analyzed by using chi-square and logistic regression to determine factors associated with prevalence of GBS among pregnant women and newborns. Descriptive statistics was used to determine prevalence of GBS among pregnant women and newborns. *P* value less than 0.05 was considered statistically significant.

**Result:**

Prevalence of GBS among pregnant women, newborns and vertical transmission rate at Hawassa University Comprehensive Specialized Hospital were 44(15.7%), 26(8.9%) and 59.1% respectively. Among 26 GBS colonized newborns one developed sign and symptoms of early onset disease. Serotype distribution of GBS isolates collected from pregnant women and newborns was Ia 13(18.6%), Ib 9(12.9%), II 24(34.3%), III 8(11.4%), V 14(20%), and NT 2 (2.9%).

**Conclusion:**

In our study we found relatively high prevalence of GBS among pregnant women and vertical transmission rate. The most prevalent GBS serotypes identified in this study were serotype II followed by V, Ia and Ib. Therefore, appropriate prevention strategies such as intrapartum antibiotic prophylaxis and vaccine development should be considered.

## Background

Neonatal disease caused by GBS is traditionally classified as early onset disease (EOD) which occurs in newborn less than 7 days of age and late onset disease (LOD) which occurs in newborn whose age is between 7 days to 90 days. The primary risk factor for EOD is recto vaginal colonization of pregnant women with GBS before delivery [1, 2]. Prevalence of GBS among pregnant women in the world is estimated to be 18% with regional variation of 11–35% [3]. Risk factors such as prolonged rupture of membrane, prematurity, chorioamnionitis, low-level of anti-GBS capsular antibody and previous newborn with EOD can increase the likelihood of EOD [1–3].

Intrapartum antibiotic prophylaxis (IAP) strategy is being used in some industrialized countries to prevent vertical transmission of GBS from pregnant women to their newborns. The strategy recommends administration of IAP based on universal screening of pregnant women for GBS at 35–37 weeks of gestation or those with risk factors such as preterm labor or premature rupture of membranes; prolonged rupture of membranes; intrapartum fever ≥100.4°F; history of a previous newborn disease caused by GBS and GBS bacteruria during pregnancy [4].

Penicillin is the drug of choice for prophylaxis, and ampicillin is an alternative [5]. For pregnant women who are allergic to penicillin and without a history of anaphylaxis, cefazolin is the preferred antibiotic. Vancomycin is recommended for those with a history of anaphylaxis, if GBS is resistant to erythromycin and clindamycin. Erythromycin or clindamycin can be used in some countries for IAP, if GBS are susceptible to them. However because of high resistance erythromycin is no longer recommended in the United States for pregnant women who are allergic to penicillin [6].

The prevention strategy does not eliminate all cases of EOD ceased by GBS; it does not affect LOD caused by GBS and there is concern of selection of antimicrobial resistant bacteria [3]. Above all, it is not feasible for developing countries including Ethiopia, with resource limitation in laboratory diagnosis. As an alternative, capsular based vaccine is being developed and currently vaccine formulation containing Ia, Ib, and III has completed phase II clinical trial and has been reported to be cost effective [4, 7]. To come up with comprehensive vaccine, data on GBS serotype is required from various geographic locations.

There is limited information regarding prevalence of GBS among pregnant women and newborns, vertical transmission rate, and serotype distribution in Southern parts of Ethiopia. Therefore, this study was conducted with the aim of determining prevalence of GBS among pregnant women and their newborns, vertical transmission rate of GBS from pregnant women to their newborns, serotype distribution of GBS and risk factors associated with prevalence of GBS among pregnant women and their newborns attending Hawassa University Comprehensive Specialized Hospital.

## Methods

### Study design

A Hospital based cross-sectional study was conducted from November 05, 2014 to March 25, 2015 at Hawassa University Comprehensive Specialized Hospital, Hawassa, Ethiopia.

### Study area

**Fig. 1:**
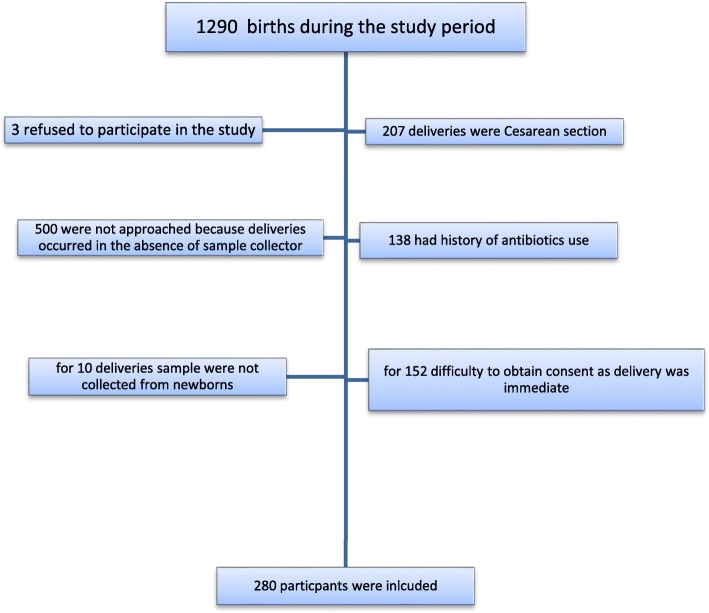
Recruitment of study participants at Hawassa University Comprehensive Specialized Hospital, Hawassa, Ethiopia, November 2014–March 2015

Hawassa is the capital city of South nation and nationalities region of Ethiopia on the shore of Lake Hawassa and it is located 275 km south of Addis Ababa, capital city of Ethiopia. The hospital is the largest hospital in the region; it serves as teaching, training and clinical service center. The Antenatal clinic of Hawassa University Comprehensive Specialized Hospital serves an average of 45 pregnant women per day and about 200 beds are available for both prenatal and postnatal service in the hospital. During the study period there were 1290 deliveries out of this 280 participants were included in the study, the rest were not included in the study due to various reasons (Fig. 1). Institutional delivery in the study area was 25.5% (information was obtained from health management information system of Hawassa Comprehensive Specialized Hospital.)

### Study population

All pregnant women admitted for delivery at Hawassa University Comprehensive Specialized Hospital during the study period who fulfilled the inclusion criteria and provided informed consent were screened for GBS colonization. Convenience sampling technique was used to recruit the study participants. Newborns were followed for sign and symptoms of EOD through telephone for 7 days. Telephone interview was made with parents about their newborns health status by using ten questions that could indicate disease development. Sample size was calculated by using single proportion formula, margin of error = 0.05, confidence interval = 95%, prevalence from previous study conducted in Ethiopia, 20.86% [8].

### Inclusion criteria

Pregnant women of all gestational age who were admitted to Hawassa University Comprehensive Specialized Hospital and were able to provide consent.

### Exclusion criteria

Pregnant women who were on antibiotics for the last 3 weeks, pregnant women with cesarean section delivery.

### Operational definition

Early onset disease: Newborn (age less than 7 days) with fever, hypothermia, vomiting, poor appetite, abnormal breathing, buldged anterior fontanelle as interviewed by telephone.

Premature rupture of membrane: rupture of membrane which occurs at gestation age less than 37 weeks.

Chorioamnionitis: Pregnant women with intrapartum fever, uterine tenderness, fetal tachycardia, maternal tachycardia, and foul smelling or purulent amniotic fluid.

Vertical transmission rate: Transmission of GBS from pregnant women to their newborns as confirmed by isolating the same colonizing GBS serotype from both mother and newborn.

### Sample collection, handling and transport

Swab from lower vaginal and rectal area of pregnant women and swabs from different body parts of newborns were collected by attending midwifery. Two specimens were collected by rolling separate sterile swabs over lower vagina and the rectal region of pregnant women, according to CDC guideline [6]. Specimens were collected from newborns by rubbing external ear, nasal area, throat and umbilical area by using sterile swabs. All specimens were collected by attending midwifery from informed and consented pregnant women during delivery. All collected specimens were placed in Stuart transport media (BD Diagnostics, USA) and was delivered to the Microbiology Laboratory within 4 h.

### Culture and identification of GBS

Vaginal and rectal swabs from pregnant women during delivery and swabs from external ear, nasal, throat and umbilical area of newborns were placed into Lim broth (BD Diagnostics, USA) supplemented with colistin (10 μg/ml) and nalidixic acid (15 μg/ml). The inoculated selective medium was incubated for 18–24 h, at 37 °C in CO_2_ enriched atmosphere, sub-cultured onto sheep blood agar (BD Diagnostics, USA) and further incubated in CO_2_ enriched atmosphere at 37 °C for 18–24 h. If GBS was not identified after incubating for 18–24 h, the blood agar plate was re-incubated and examined after 48 h to identify suspected colonies. All suspected GBS colonies (β-hemolytic, or non-hemolytic, gram positive cocci, catalase negative) were sub-cultured and isolated for confirmatory testing. A CAMP (Christite, Atkins, Munch, Petersen) test were considered presumptive identification of a positive GBS culture. Ambiguous CAMP test culture results were re-tested using a Strpt. B Grouping Latex (Remel, USA).

Capsular serotyping was done for all GBS isolates by slide agglutination tests by using type specific 10 antisera for serotypes Ia, Ib, II, III, IV, V, VI, VII, VIII and IX (Statens Serum Institute, Denmark) as previously described by Slotved et al [9].

### Quality control

To maintain the quality of culture medias and reagents, control strains *Staphylococcus aureus* (ATCC 24923*), Streptococcus pyogenes* (ATCC 19615), and *Streptococcus agalactiae* (ATCC12403) were used during the study. To maintain the quality of antisera, the manufacture’s instruction was followed, and positive and negative controls were used. For socio-demographic, obstetric and clinical data a pretested structured questionnaire was used and data was entered in two separate computers. To ensure the viability of GBS, all samples were placed in transport media immediately after collection and processed within 4 h of collection.

### Statistical analysis

Data entry and analysis was done using computer with SPSS version 20 software. Frequency distribution was used to calculate prevalence figures from the total study population and separately by age group and risk factors. Bivariate logistic regression was used to compare prevalence of GBS with various factors. *P* value less than 0.05 was considered significant.

## Results

### Socio-demographic characteristics

Among 1290 deliveries that occurred during the study period, 280 pregnant women along with their 292 newborns participated in the study. 147(52.5%), 40(14.3%) and 93(33.2%) participants were from Hawassa, Shashamane and other areas respectively. 232(82.9%) participants were housewives and 111(39.6%) participants belongs to the age group of 21–25 years. Out of 292 newborns 152(52.1%) were males and 140(47.9%) were females.

### Obstetric characteristics

#### Pregnant women

Out of the total 280 pregnant women participating in the study, 249(88.9%) delivered at gestational age of 37–42 weeks; 134(47.9%) of participants were primigravida and 146 (52.1%) were multigravida, 115(41.1%) participants had previous vaginal delivery; 241(86.4%) had ruptured membrane of 0–5 h duration and 18(6.4%) were with premature rupture of membrane (Table 1).

**Table 1:** Obstetrics characteristics of pregnant women attending Hawassa University Comprehensive Specialized Hospital, November 2014–March 2015 (*n* = 280)

Characteristics		Frequency	Percentage (%)
Gestational age in weeks	< 37	25	8.9
	37–42	249	88.9
	> 42	6	2.1
Gravida	Primigravida	134	47.9
	Multigravida	146	52.1
Previous infant with EOD	No	146	52.1
	NA	134	47.9
Mode of delivery	VD	115	41.1
	CS	30	10.7
	NA	134	47.9
	VD and CS	1	0.4
Duration of membrane rupture	0-5 h	241	86.1
	6–10	15	5.4
	11–15	10	3.60
	16–20	1	0.40
	21–25	4	1.40
	> 25	9	3.20
Premature rupture of membrane	Yes	18	6.4
Chorioamnionitis	Yes	2	0.7
	No	278	99.3
Meconium stained amniotic fluid	Yes	36	12.33
	No	244	87.14
Other illness	Yes	11	3.9

*NA* not applicable, *EOD* early onset disease, *CS* cesarean section, *VD* vaginal delivery

#### Newborns

Among 292 newborns participating in the present study, 12(4.1%) were twins and 280(95.9%) were alive at birth, 248(84.9%) were in the weight range of 2500-4000 g, 161 (55.1%) had APGAR score at 5 min > 7; and those with other abnormalities were 13(4.5%). All newborn twins were alive at birth (Table 2).

**Table 2:** Clinical characteristics of newborns delivered at Hawassa University Comprehensive and Specialized Hospital, November 2014–March 2015 (*n* = 292)

Characteristics		Frequency	Percentage (%)
	Yes	12	4.1%
Status of newborn at birth	Alive	280	95.9
	Dead	12	4.1
Weight	< 1500 g	4	1.4
	1500-2499 g	28	9.6
	2500-4000 g	248	84.9
	> 4000	12	4.1
APGAR score at 5 min	< 7	131	44.9
	> 7	161	55.1
APGAR score at 10 min	< 7	35	12
	> 7	257	88
Developed EOD	Yes	5	1.7
Other abnormality^a^	Yes	13	4.5

*APGAR* appearance, pulse, grimace, activity, respiration, *EOD* early onset disease

^a^Other disease include cyanosis, spinal bifida, hydrocephaly

### Prevalence of GBS among pregnant women and their newborns and vertical transmission rate

The prevalence of GBS among pregnant women and their newborns and vertical transmission rate were 44/280(15.7%), 26/292(8.9%), and 26/44(59.1%) respectively. The serotype distribution of GBS isolated from pregnant women and newborns was: Ia 13(18.6%), Ib 9(12.9%), II 24(34.3%), III 8(11.4%), V 14(20%), and NT 2(2.9%). All GBS serotypes identified from newborns were the same as GBS serotypes identified from their respective mothers (Table 3). Out of 26 newborns colonized with GBS, 1 developed signs and symptoms of EOD.

**Table 3:** Serotype distribution of GBS isolated from pregnant women and their newborns and vertical transmission rate at Hawassa University Comprehensive Specialized Hospital, November 2014–March 2015 (*n* = 70)

Serotype	Pregnant women	Newborns	Total	VTR
Ia	9/44 (20.5%)	4/26 (15.4%)	13/70 (18.6%)	4/9 (44.4%)
Ib	5/44 (11.4%)	4/26 (15.4%)	9/70 (12.9%)	4/5 (80%)
II	14/44 (31.8%)	10/26 (38.5%)	24/70 (34.3%)	10/14 (71.4%)
III	6/44 (13.6%)	2/26 (7.7%)	8/70 (11.4%)	2/6 (33.3%)
V	8/44 (18.2%)	6/26 (23.1%)	14/70 (20%)	6/8 (75%)
NT	2/44 (4.6%)	0/26 (0)	2/70 (2.9%)	0/2 (0)
Total	44	26	70	26/44 (59.1)

*VTR* vertical transmission rate, *NT* non typeable, I, II,I II.V=Serotypes

Out of the 12 newborn twins, 2 (one pair) were colonized with GBS. Among the 12 newborns who were not alive at birth, 2 were colonized with GBS. Out of 44 mothers colonized with GBS, 2 gave birth to babies who were not alive at birth, and out of 236 mothers who were not colonized with GBS, 10 had newborns who were not alive at birth.

### Factors associated with prevalence of GBS among pregnant women and newborns

#### Pregnant women

The prevalence of GBS among pregnant women and their newborns was not significantly associated with the majority of factors. Newborns with other disease had 4.95 time chance to be colonized with GBS (Tables 4 and 5). Other disease includes cyanosis, spinal bifida, and hydrocephaly.

**Table 4:** Bivariate analysis of risk factors associated with prevalence of GBS among pregnant women, at Hawassa University Comprehensive Specialized Hospital, November 2014–March 2015 (*n* = 280)

Variables		Prevalence of GBS	OR(95%CI)	*P*-value
Age group	15–27	28/190 (14.7%)	0.79 (0.41–1.56)	0.51
	> 28	16/90 (17.8%)	1	
Gestational age	< 37	4/25 (16%)	0.9 (0.08–10.5)	0.9
	37–42	38/249 (15.3%)	0.93 (0.1–8.1)	0.95
	> 42	2/6 (33.3%)	1	
Gravida	Primigravida	24/134 (17.9%)	1.38 (0.7–2.6)	0.34
	Multigravida	20/146 (13.7)	1	
History of EOD	No	20/146 (13.7%)	0.73 (0.38–1.4)	0.34
	NA	24/134 (17.9%)	1	
Mode of delivery	Vaginal	16/115 (13.9%)	0.69 (0.34–1.38)	0.29
	CS	4/31 (12.9%)	0.88 (0.31–2.53)	0.84
	NA	24/134 (17.9%)	1	
Duration of rupture of membrane	0–5 h	39/241 (16.2%)	2.1 (0.47–9.1)	0.34
	6–10 h	4/15 (26.7%)	4 (0.6–25.3)	0.14
	11–15 h	1/24 (4.2%)	1	
Meconium stained amniotic fluid	Yes	4/36 (11.1%)	0.85 (0.3–2.3)	0.75
	No	40/244 (16.4%)	1	
Other illness	Yes	2/11 (18.2%)	1.2 (0.25–5.7)	0.82
	No	42/269 (15.6%)	1	

*N* total number of pregnant women, *OR* odds ratio, *GBS* group B streptococcus, *CS* caesarian section, *NA* not applicable

**Table 5:** Bivariate analysis of risk factors associated with Newborn prevalence of GBS, Hawassa University Comprehensive Specialized Hospital, November 2014–March 2015 (*n* = 280)

Variables		Prevalence of GBS,	OR(95%CI)	*P*-value
Weight	1000-2499 g	3/32 (9.4%)	1.5 (0.27–9.11)	0.9
	2500-4000 g+	23/260 (8.8%)	1	
APGAR score at 5 min	< 7	11/131 (8.4%)	0.83 (0.37–1.9)	0.65
	> 7	15/161 (9.3%)	1	
Week of birth	< 37	3/28 (10.7%)	0.6 (0.05–7)	0.68
	37–42	22/258 (8.5%)	0.47 (0.05–4.37)	0.55
	> 42	1/6 (16.7%)	1	
APGAR score at 10 min	< 7	5/35 (14.3%)	1.78 (0.62–5)	0.28
	> 7	21/257 (8.6%)	1	
Status of newborns at birth	Dead	2/12 (16.7%)	2.0 (0.42–9.8)	0.37
	Alive	24/280 (8.6%)	1	
Developed EOD	Yes	1/5 (20%)	2.5 (0.27–23.3)	0.42
	No	25/287 (8.7%)	1	
Other disease^a^	Yes	4/13 (30.8%)	4.95 (1.41–17.3)	0.012
	No	22/279 (7.9%)	1	

*N* total number of newborn, *OR* odds ratio, *GBS* group B Streptococcus, *EOD* early onset delivery, *APGAR* appearance, pulse, grimace, activity, respiration

^a^Other disease include cyanosis, spinal bifida, hydrocephaly

## Discussion

In this study, prevalence of GBS among pregnant women and newborns and vertical transmission rate from pregnant women to their newborns at Hawassa University Comprehensive Specialized Hospital, Ethiopia was 15.7, 8.9 and 59.1% respectively. Prevalence of GBS among pregnant women in this study was comparable with prevalence of GBS among pregnant women reported from Brazil 14.6% and Germany 16% [10, 11]. In contrast to our finding, some countries in Asia reported low prevalence of GBS among pregnant women [12–14]. Studies from South Africa, Europe and recent systematic review and meta-analysis reported higher prevalence of GBS among pregnant women than current study [2, 3, 6, 15]. Similarly, a previous study from Hawassa, Ethiopia reported high prevalence of GBS among pregnant women (20.8%) [8], while comparable result was reported from central part of Ethiopia, Addis Ababa (14.6%) [16] and western parts of Ethiopia (12.2%) [17, 18]. Unlike this study, a higher maternal GBS colonization rate was reported from Nigeria (64%) [19].

These results indicate that the prevalence of maternal GBS colonization, which is primary risk factor for EOD, differs in different countries and within the same country [3]. The difference can be due to geographical differences, laboratory methods used, time and site of specimen collection [3, 14, 15, 20]. The prevalence of GBS among newborns, 8.9%, and vertical transmission rate of GBS from pregnant women to their newborns, 59.1% identified in this study were comparable with the report from earlier studies conducted in the 1970s [21, 22]. Previous studies from developed countries indicated that about 50% newborns from GBS colonized pregnant women will be positive for GBS at birth. Among GBS colonized newborns, approximately 1–3% will develop invasive disease [22]. Vertical transmission rate of GBS from mother to newborns reported from eastern part of Ethiopia, 45.02%, was lower compared to the current study [23]. The difference observed can be due to several reasons such as sample collection, laboratory methods and factors related to study participants.

Five newborns in our study developed sign and symptoms of EOD and four were from 287 GBS non-colonized newborns and one was from 26 GBS colonized newborns. One newborn who developed sign and symptoms of EOD was colonized with GBS and the mother was also positive for GBS, this may indicated GBS was transferred from mother to newborn and was responsible for the development of EOD even though the causative agent was unconfirmed. In this study, out of 26 GBS colonized newborns two (7.7%) were not alive at birth. There was no difference in prevalence of stillbirth among GBS colonized mothers two (4.5%) and non GBS colonized mothers 10/236(4.2%) (*P* = 0.63); this may be due to small sample size. Among 12 mothers with stillbirth, only one showed signs and symptom of disease. Even though it is not well established, some studies indicated maternal GBS colonization could cause still birth [24]. Systematic review and meta-analysis by Seale et al [25] estimated that 1% of all stillbirth in developed countries and 4% in Africa were associated with GBS. In our study, we did not find significant association between prevalence of GBS among pregnant women and newborns and majority of factors measured (*P* > 0.05). The only factor that showed significant association with neonatal GBS colonization was newborn who presented with other disease (*P* = 0.012).

There is scarce or no data on GBS serotype distribution from the study area. In the present study, the most prevalent serotypes was serotype II followed by serotype V (20%), Ia (18.6%), Ib 12.9%, and III (11.4%). Out of the total GBS strains collected, 2.9% were non-typeable.

Group B streptococcus (GBS) serotype distribution is not uniform across different countries [20]. The prevalence of serotype II in this study was high compared to report from several countries, which is less than 15% [3, 4, 26]. Relatively higher prevalence of serotype II (19.1%) was reported from Brazil [27], even though it is still far below the finding of our study. Serotype V was the second most prevalent in our study area, in line with what was reported in USA (17%) [28]. In contrast to this study, a low prevalence of serotype V was report in Japan (12%) [29], Poland (5%) [30], Brazil (13.6%) [27], and China (14.2%) [13].

Prevalence of serotype Ia detected in this study was similar with prevalence of serotype Ia reported from China (17.9%) [13] and Malaysia (17.5%) [26]. Higher prevalence of serotype Ia was reported from Brazil (27.6%) [27] and Nigeria (23.9%) [19]. Even though serotype Ib was the third most prevalent GBS serotype in this study, it was high compared to reports from many countries such as Poland (5%) [30] and South Africa (6.7%) [31]. Unlike the present study, high prevalence of serotype Ib was reported from China (16.1%) [13].

The least prevalent serotype found in the current study was serotype III. In contrast to the current study higher prevalence of serotype III was reported from Poland (60%) [30], Korea (29.8%) [32] and China (32.1%) [13]. Several studies have indicated that GBS serotype III to be the most virulent compared to other serotypes [10].

The few numbers of GBS isolates which were non-typeable does not allow comparison with other findings with on-typeability rate reported from other countries such as 2% from South Africa [33] and 20% from Brazil [34]. There are several explanations for non-typeability of GBS such as; non-encapsulated variant, uncharacterized polysaccharide, mutation in genes essential for capsule expression, reversible non-capsular phase variation or technical problem [35].

Serotype Ia, Ib, II, III and V are frequently mentioned in Africa including in our study, while serotypes IV, VI, VII, VIII, and serotype IX were rarely reported [10]. However, Cools et al. [36] reported relatively high prevalence of serotypes VI, VII, and VIII in Kenya and serotypes IV, VI and VIII in South Africa, but serotype Ib was not detected.

Based on our study vaccine formulation containing serotype II, V, Ia, Ib and III may prevent EOD caused by GBS in the study area. The current vaccine formulation in clinical trial, which is composed of Ia, Ib and III, may not cover all the GBS serotypes circulating in the study area, particularly the major serotypes, II and V which are missing from the vaccine formulation. Therefore, it is important to have data on GBS serotypes from different regions to develop a universal vaccine.

Limitations of this study: we were unable to confirm the causative agent of EOD and stillbirth by using culture method and we were unable to confirm the causative agent of stillbirth. As we used convenience sampling technique selection bias was not avoided and the study population was not representative of all pregnant women in the study area. We used this method of sampling technique because the time and budget allocated for the study was limited. Risk factors such as sexual transmitted disease and socioeconomic status were not measured.

## Conclusion

Prevalence of GBS among pregnant women and newborns and vertical transmission of GBS from pregnant women to their newborns at Hawassa University Comprehensive Specialized Hospital were 15.7, 8.9 and 59.1% respectively. The predominant GBS serotypes we found in this study were II, V, Ia, Ib, and III. Majority of the risk factors measured did not show significant association with prevalence of GBS among pregnant women and newborns. Based on the findings of the current study we recommend that appropriate prevention strategy should be implemented to reduce neonatal disease that can be caused by GBS in the study area. GBS serotypes which are prevalent in the study area need to be considered during vaccine development. Most importantly, future studies in Ethiopia should focus on measuring the burden of EOD and LOD caused by GBS.

## Abbreviations

APGAR: Appearance, pulse, grimace, activity, respiration
CDC: Center of Disease Control and Prevention
EOD: Early onset disease
GBS: Group B streptococcus
HUSH: Hawassa University Comprehensive Specialized Hospital
IAP: Intrapartum antibiotic prophylaxis
LOD: Late onset disease
PROM: Premature rupture of membrane
